# Vestibular Function in Children and Adults Before and After Unilateral or Sequential Bilateral Cochlear Implantation

**DOI:** 10.3389/fneur.2021.675502

**Published:** 2021-04-29

**Authors:** Ruirui Guan, Yanqi Wang, Sasa Wu, Bo Zhang, Jingwu Sun, Xiaotao Guo, Jiaqiang Sun

**Affiliations:** Department of Otolaryngology Head and Neck Surgery, The First Affiliated Hospital of USTC, Division of Life Sciences and Medicine, University of Science and Technology of China, Hefei, China

**Keywords:** cochlear implant, vestibular function, children, adults, unilateral implantation, sequential bilateral implantation

## Abstract

**Background:** Cochlear implantation (CI) helps patients with severe or profound sensorineural hearing loss (SNHL) restore hearing and speech abilities. However, some patients exhibit abnormal vestibular functions with symptoms such as dizziness or balance disorders, after CI. Whether age at CI and CI approach (unilateral or sequential bilateral) affect vestibular functions in users with cochlear implants remains unclear.

**Objectives:** To investigate the vestibular functions in children and adults before and after unilateral or sequential bilateral CI.

**Materials and Methods:** Thirty-seven patients with severe or profound SNHL who were candidates for a first- or second-side CI were divided into three groups: first-side CI-implanted adults (≥18 years), first-side CI-implanted children (6–17 years), and second-side CI-implanted children (6–17 years). All cases were implanted with the round window approach to minimize damage to the intra-cochlear structures. The caloric test, vestibular evoked myogenic potential (VEMP) test, video head impulse test (vHIT), Dizziness Handicap Inventory (DHI), Pediatric Vestibular Symptom Questionnaire (PVSQ), and audiometric tests were performed before and 1 month after CI.

**Results:** The abnormal rates of caloric test and VEMP test after CI in the first-side CI-implanted adults and children significantly increased compared with those before CI. The pre-implantation VEMP test showed significantly higher abnormal rates between first- and second-side CI-implanted children. No other significant differences of abnormal rates between first- and second-side CI-implanted children or between first-side CI-implanted adults and children were found. In second-side CI-implanted children, PVSQ scores significantly increased at day 3 post-implantation but decreased at day 30.

**Conclusion:** CI has a negative effect on the results of caloric and VEMP tests, but not on vHIT, indicating that the otolith and low-frequency semicircular canal (SCC) are more vulnerable to damage from CI. The alterations of vestibular functions resulting from CI surgery may be independent of age at CI and CI approach (unilateral or sequential bilateral). Long-term impacts on the vestibular function from CI surgery, as well as the chronic electrical stimulation to the cochlea, are still to be investigated.

## Introduction

Cochlear implantation (CI) is successfully used as a treatment option for restoring hearing in patients with severe or profound sensorineural hearing loss (SNHL). While CI restores hearing, in some patients, it negatively affects vestibular functions, resulting in dizziness, or balance disorders. In 1971, Michelson first reported that a CI patient developed vertigo and tinnitus symptoms after CI surgery (1). Terry et al. analyzed delayed complications after CI in a review capturing 88 studies and found that vestibular complications were the most common phenomenon (2). Sokolov et al. reported that deaf children are more likely to have vestibular disorders (3). Modifications and areflexia of vestibular functions have been previously reported (4). The CI electrode stimulates the spiral ganglion in the central modiolus trunk, which affects the stability of the endolymphatic environment, possibly resulting in histopathological changes after CI.

With the popularization of CI, vestibular dysfunction has attracted much attention recently. Most studies, however, have focused on vestibular functions in adults rather than in children because of the often-poor co-operation and unreliable responses of children during testing. The effect of CI on vestibular functions in children remains unclear. The advantages of binaural hearing through bilateral CI are widely accepted, and more patients choose bilateral CI. Simultaneous bilateral implantation may cause more risks to patients compared with sequential bilateral implantation from the surgical point of view (5). But considering the health economics and the process of learning to use CI, simultaneous bilateral CI is considered better (6). Clinical studies showed that the incidence of vertigo after CI was 2–35%, but the incidence of vestibular dysfunction was 20–80% (5, 7, 8). The results of subjective and objective tests for evaluating vestibular function seem inconsistent.

Obviously, the incidence of vestibular dysfunction after CI changes greatly, and the reasons are multifactorial. In this study, to investigate the effects of age at CI and CI approach (unilateral or sequential bilateral) on vestibular functions, we evaluated vestibular functions of five vestibular end organs (saccule, utricle, and semicircular canals) in children and adults before and after unilateral or sequential bilateral CI.

## Materials and Methods

### Patients

In this study, 37 patients (23 males and 14 females) with bilateral severe or profound SNHL receiving CI in our hospital from October 2020 to January 2021 were included. No patients had inner ear malformations and all had normal eardrums and middle ear pressures before and 1 month after CI. Patients had no pathological features of the vestibular, oculomotor or neuromuscular system except for one adult patient with Meniere's disease. Children in this study were more than 6 years old. They had good comprehension and expression abilities and could clearly describe their vertigo symptoms. Each patient was implanted with a 28 mm long electrode array (FLEX28) through the round window (RW) approach to minimize damage to the intra-cochlear structures. The implanted devices were produced by MED-EL (Innsbruck, Austria). No serious complications after CI surgery were found. Each patient underwent audiometric tests (tympanometry and pure tone audiometry) and a series of vestibular function tests 1–3 days before surgery and 29–37 days after surgery. The vestibular function tests included the caloric test, vestibular evoked myogenic potential (VEMP) test, video head impulse test (vHIT), Dizziness Handicap Inventory (DHI) for adults, and Pediatric Vestibular Symptom Questionnaire (PVSQ) for children.

Patients were divided into three groups according to the age at implantation and the first- or second-side CI: first-side CI-implanted adults (≥18 years, *n* = 15), first-side CI-implanted children (6–17 years, *n* = 10), and second-side CI-implanted children (6–17 years, *n* = 12). First-side CI-implanted adults and children received a unilateral CI, and second-side CI-implanted children received a sequential bilateral CI. The mean (±SD) ages of first-side CI-implanted adults, first-side CI-implanted children, and second-side CI-implanted children were 37.46 ± 15.32 years (ranging from 18 to 70 years), 10.00 ± 4.24 years (ranging from 6 to 16 years), and 8.92 ± 4.11 years (ranging from 6 to 17 years), respectively. The pure tone averages (PTAs) at 500 Hz, 1, 2, and 4 kHz for all patients were 102.3 ± 13.65 dB HL (left side) and 102.1 ± 20.89 dB HL (right side), ranging from 75 to 120 dB HL. The demographic information of patients is listed in Table 1. The protocols and experimental procedures in the present study were reviewed and approved by the Anhui Provincial Hospital Ethics Committee. Each participant or his/her guardians had filled out informed consent carefully before the experiment.

**Table 1:** The demographic information of participants with cochlear implants.

**Subject**	**Gender**	**Etiology**	**Age at first/second CI (years)**	**First/second implanted side**	**First/second Implant type**
**First-side CI-implanted adults**					
1	M	Hereditary	22/	R/	SONATATi100
2	M	Unknown	33/	L/	SONATAtTi100
3	F	Unknown	18/	R/	CONCERTOMi1000
4	M	Meniere's disease	70/	R/	CONCERTOMi1000
5	F	Unknown	56/	R/	CONCERTOMi1000
6	M	Unknown	19/	R/	SONATATi100
7	M	Noise induced	46/	R/	CONCERTOMi1000
8	F	Unknown	40/	L/	SONATATi100
9	M	Noise induced	44/	L/	CONCERTOMi1000
10	F	Sudden deafness	48/	L/	CONCERTOMi1000
11	M	Unknown	20/	R/	CONCERTOMi1000
12	F	Unknown	43/	R/	CONCERTOMi1000
13	M	Drug-induced	24/	R/	CONCERTOMi1000
14	F	Sudden deafness	48/	R/	CONCERTOMi1000
15	M	Unknown	31/	L/	SONATATi100
**First-side CI-implanted children**					
1	M	Unknown	16/	R/	CONCERTOMi1000
2	M	Hereditary	6/	L/	CONCERTOMi1000
3	F	Unknown	8/	R/	SONATATi100
4	F	Unknown	6/	R/	CONCERTOMi1000
5	M	Hereditary	8/	L/	CONCERTOMi1000
6	M	Viral infection	13/	R/	SONATATi100
7	M	Hereditary	16/	L/	CONCERTOMi1000
8	M	Viral infection	6/	R/	CONCERTOMi1000
9	F	Unknown	14/	L/	SONATATi100
10	M	Viral infection	7/	R/	CONCERTOMi1000
**Second-side CI-implanted children**					
1	F	Unknown	1/6	R/L	SONATAti100/CONCERTOMi1000
2	M	Viral infection	9/12	L/R	CONCERTOMi1000 (Bi)
3	M	Drug-induced	1/6	R/L	SONATAti100 (Bi)
4	M	Unknown	1/6	L/R	SONATAti100 (Bi)
5	M	Unknown	10/16	R/L	CONCERTOMi1000 (Bi)
6	F	Hereditary	3/8	L/R	CONCERTOMi1000 (Bi)
7	F	Unknown	3/6	R/L	CONCERTOMi1000 (Bi)
8	M	Viral infection	10/11	R/L	CONCERTOMi1000 (Bi)
9	F	Unknown	2/9	R/L	CONCERTOMi1000 (Bi)
10	M	Unknown	1/7	R/L	SONATAti100 (Bi)
11	M	Unknown	2/8	R/L	SONATAti100/CONCERTOMi1000
12	M	Hereditary	16/17	R/L	SONATAti100 (Bi)

*CI, cochlear implant; F, female; M, male; L, left; R, right; Bi, bilateral*.

### Vestibular Function Tests

#### Caloric Test

The caloric test was employed to evaluate the horizontal semicircular canal (SCC). The patients took supine position and raised their head to 30 degrees with a pillow. The right and left ears of the patients were stimulated with cool air (24°C) and warm air (50°C) by using an air caloric irrigator system (Micromedical Technologies Inc., Chatham, IL, USA). We used videonystagmography (VNG) (VisualEyesTM VNG, Micromedical Technologies Inc., USA) to record horizontal eye movements during the test. The subjects were perfused four times for 60 s. After perfusion, the nystagmus was observed for 60 s. Unilateral Weakness (UW) was calculated using the maximal slow phase eye velocity: UW = | (RC + RW) – (LC + LW) | / (RC + RW +LC + LW) × 100%, where RC = right cool, RW = right warm, LC = left cool, and LW = left warm. UW >25% was considered abnormal (9).

#### VEMP

VEMP was recorded by using a SmartEP equipment (Intelligent Hearing Systems, Miami, FL, USA). All patients were tested with air-conducted tone bursts of 105 dB nHL at 500 Hz administered by plug-in earphones. The resistance of the electrode was <5 kΩ. For ocular VEMP (oVEMP), the first negative waveform is N1 (at the latency of about 10 ms) and the first positive waveform is P1 (at the latency of about 15 ms). For cervical VEMP (cVEMP), the first positive waveform is P1 (at the latency of about 13 ms) and the first negative wave is N1 (at the latency of about 23 ms). Whereas, oVEMP can be used to evaluate the function of the utricle and the superior vestibular nerve pathway, cVEMP is mainly used to evaluate the function of the saccule and the inferior vestibular nerve pathway. The amplitude asymmetry ratio (AR) was calculated as follows: | (right amplitude - left amplitude) | /(right amplitude + left amplitude) × 100%. The response was regarded as abnormal for AR >0.34 or no repeatable waveforms (10).

#### vHIT

All subjects were tested for vHIT by using the EyeSeeCam system (EyeSeeCam, Interacoustics Inc., Assens, Denmark) to record the gain of vestibulo-ocular reflex (VOR) of each SCC. The subjects took seated position and wore a light glass, which was about 1–1.5 m away from the fixed point. After the calibration according to the software requirements, three pairs of semicircular canals in the horizontal and vertical directions were tested. During the test, subjects were asked to keep their eyes fixed in front of the target and relax their necks. At least 10 impulses with peak velocity ranging from 150 to 250/s were collected from each canal. In our study, the vHIT results were classified based on the gain of vHIT. We classified abnormal values as follows: the gain of the horizontal canal (HC) was <0.8; the gain of the anterior canal (AC) or posterior canal (PC) was <0.7 (11). We measured the gains of both ears at the same time and regarded the vestibular function as abnormal if either ear showed an abnormal value.

#### Vertigo Disorders Scale Assessment

The DHI was used to evaluate the impact of dizziness or vertigo on the quality of life for adult patients. DHI includes 25 items: 9 items of emotion (E), 9 items of function (F), and 7 items of physical (P). Each item has three options, namely, “yes,” “sometimes,” and “none,” which are scored as 4 points, 2 points, and 0 points, respectively. The maximum score is 100 points. 0 points indicates that vertigo symptoms have no effect on patients. The higher the score, the more serious the impact of vertigo on patients.

The PVSQ is a measure of the severity of vestibular symptoms (dizziness, instability) in children ages 6–17 years old. There are 11 questions in this questionnaire (10 multiple-choice questions, 1 subjective question). In this study, only 10 multiple-choice questions were selected. Each question has four options. Each item is rated on a scale of 0 (never) to 3 (most of the time) (12). The answer to each item of the PVSQ was obtained from the guardians by full communication between children and guardians.

### Data Processing and Analysis

The ratio of patients with abnormal vestibular functions to total subjects in each group was regarded as the abnormal rate. Because the pre-implantation abnormal rates of vestibular functions for different groups may be different, we also assessed the growth rate which was calculated by subtracting the pre-implantation abnormal rate from the post-implantation abnormal rate. We used the SPSS software package (version 17.0 for Windows; SPSS Inc., Chicago, IL, USA) to analyze the data. Because of the small sample size, the Fisher's exact test was used to assess the differences of abnormal rates and growth rates. Among all patients, 12 first-side CI-implanted adults, 8 first-side CI-implanted children, and 9 second-side CI-implanted children completed vertigo disorders scale assessment before implantation, at day 3 after implantation, and at day 30 after implantation. The difference of DHI or PVSQ scores among three periods for each group was assessed by using a non-parametric Friedman test, and the difference of PVSQ scores between first- and second-side CI-implanted children at each period was further assessed by using a non-parametric Mann-Whitney *U*-test. If the DHI or PVSQ scores were significantly different among three periods, a Wilcoxon signed rank test was further used to assess scores between any two periods. For hypothesis testing, *p* < 0.05 was considered significantly different.

## Results

### Abnormal Rates of Vestibular Functions Before and After CI

**Figure 1:**
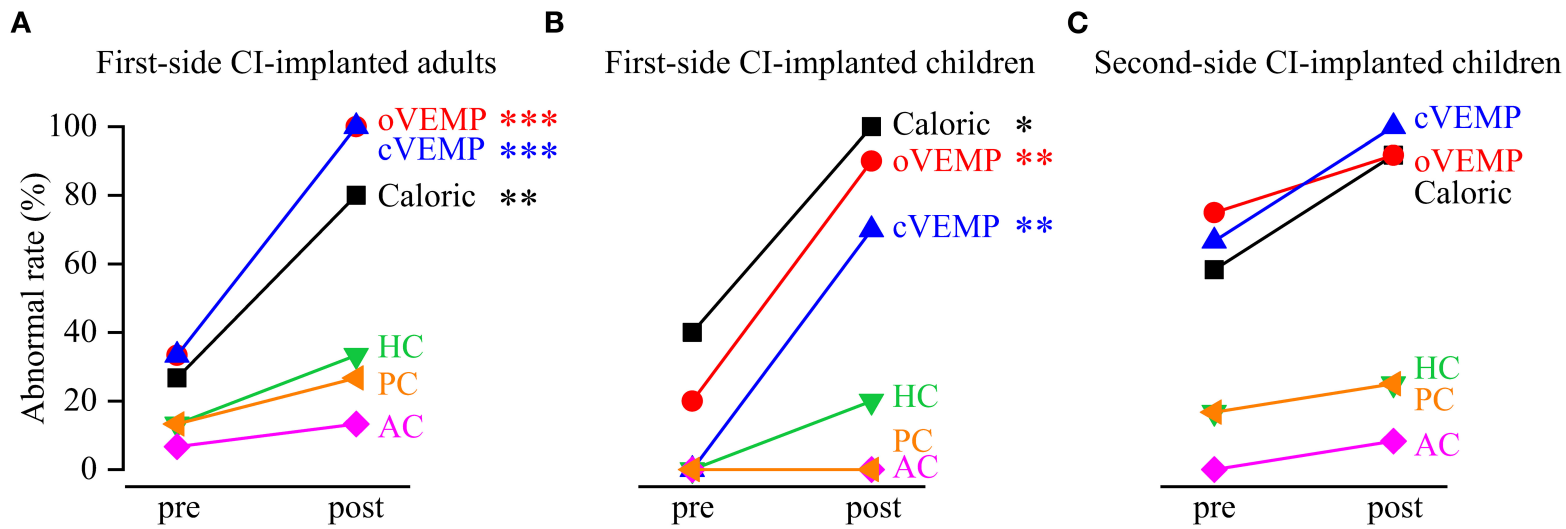
Abnormal rates of vestibular functions revealed by the caloric test, vestibular evoked myogenic potential (VEMP) test and video head impulse test (vHIT) before and after cochlear implantation (CI). The abnormal rates significantly increased from pre- to post-implantation in **(A)** first-side CI-implanted adults and **(B)** first-side CI-implanted children, as revealed by the caloric test, ocular VEMP (oVEMP) and cervical VEMP (cVEMP). The pre- and post-implantation abnormal rates of functions of horizontal canal (HC), anterior canal (AC) and posterior canal (PC) in vHIT were not significantly different. **(C)** No significant difference of the abnormal rates was found between pre- and post-implantation tests in the second-side CI-implanted children. *p < 0.05, **p < 0.01, ***p < 0.001.

The caloric test and the VEMP test showed that the abnormal rates significantly increased from pre- to post-implantation in the first-side CI-implanted adults (caloric test: pre: 26.67%, post: 80%, *p* = 0.009; oVEMP: pre: 33.33%, post: 100%, *p* < 0.001; cVEMP: pre: 33.33%, post: 100%, *p* = 0.001) and in the first-side CI-implanted children (caloric test: pre: 40%, post: 100%, *p* = 0.011; oVEMP: pre: 20%, post: 90%, *p* = 0.005; cVEMP: pre: 0%, post: 70%, *p* = 0.003) (Figure 1). For the second-side CI-implanted children, the abnormal rate was higher in post- than in pre-implantation caloric test (pre: 58.33%, post: 91.67%, *p* = 0.155) and VEMP test (oVEMP: pre: 75%, post: 91.67%, *p* = 0.590; cVEMP: pre: 66.67%, post: 100%, *p* = 0.093), but the difference was not significant. The vHIT test showed no significant difference between pre- and post-implantation abnormal rates in the first-side CI-implanted adults (HC: pre: 13.33%, post: 33.33%, *p* = 0.390; AC: pre: 6.67%, post: 13.33%, *p* > 0.999; PC: pre: 13.33%, post: 26.67%, *p* = 0.651), in the first-side CI-implanted children (HC: pre: 0%, post: 20%, *p* = 0.474; AC: pre: 0%, post: 0%, *p* > 0.999; PC: pre: 0%, post: 0%, *p* > 0.999) or in the second-side CI-implanted children (HC: pre:16.67%, post: 25%, *p* > 0.999; AC: pre: 0%, post: 8.33%, *p* > 0.999; PC: pre: 16.67%, post: 25%, *p* > 0.999).

### Abnormal Rates of Vestibular Functions Between First-Side CI-Implanted Adults and Children

**Figure 2:**
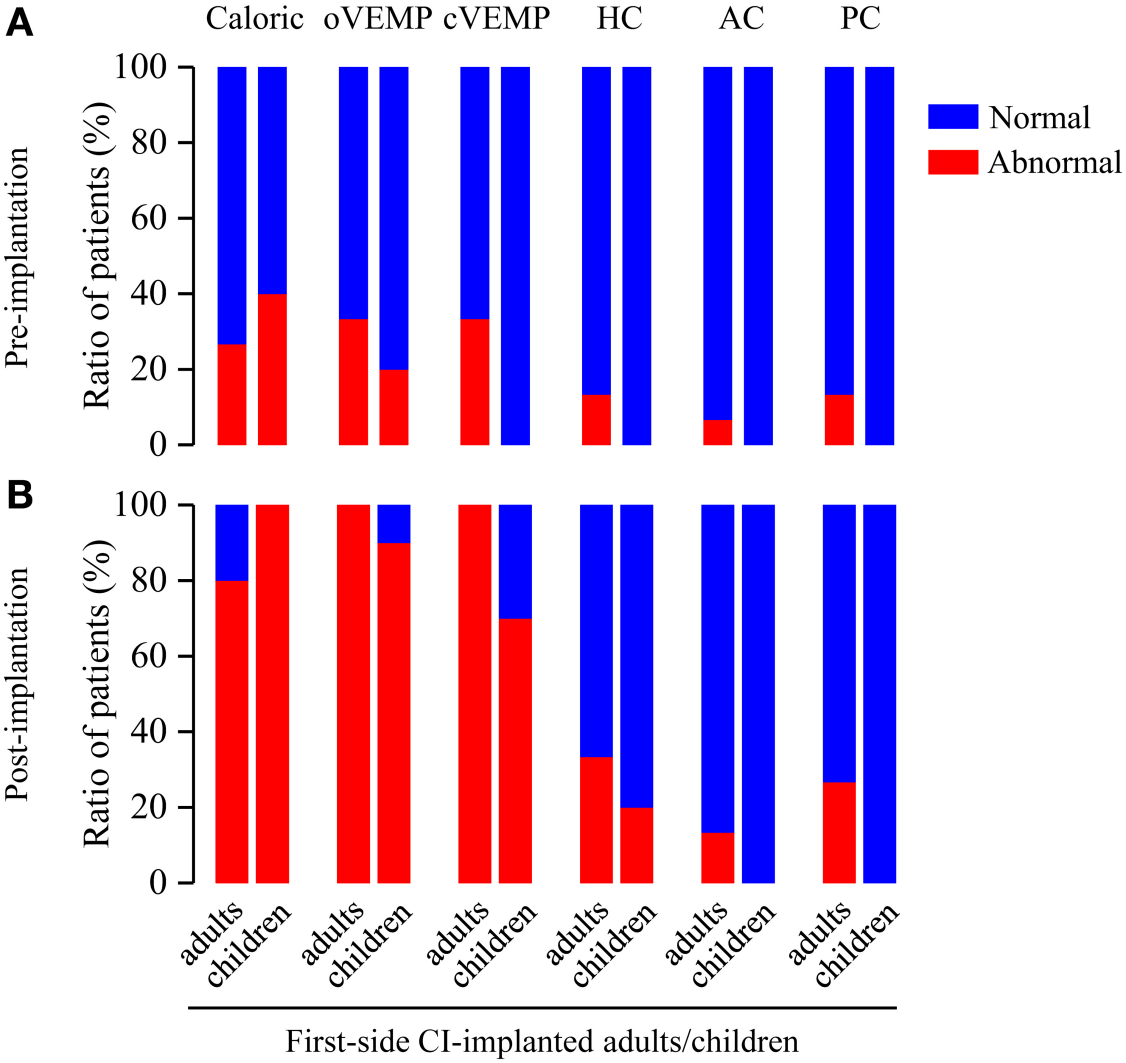
Ratio of patients with abnormal vestibular functions revealed by pre- and post-implantation caloric test, VEMP test, and vHIT in first-side CI-implanted adults and children. **(A)** Pre- and **(B)** post-implantation tests showed no significant difference of abnormal rates between first-side CI-implanted adults and children.

To assess the effect of age at CI on the vestibular functions, we analyzed the differences of abnormal rates between first-side CI-implanted adults and children. The pre-implantation caloric test, VEMP test and vHIT showed no significant difference of the abnormal rates between first-side CI-implanted adults and children (caloric test: 26.67 vs. 40%, *p* = 0.667; oVEMP: 33.33 vs. 20%, *p* = 0.659; cVEMP: 33.33 vs. 0%, *p* = 0.061; HC: 13.33 vs. 0%, *p* = 0.500; AC: 6.67 vs. 0%, *p* > 0.999; PC: 13.33 vs. 0%, *p* = 0.500) (Figure 2). Furthermore, no significant difference of the post-implantation abnormal rates between these two groups was found (caloric test: 80 vs. 100%, *p* = 0.250; oVEMP: 100 vs. 90%, *p* = 0.400; cVEMP: 100 vs. 70%, *p* = 0.052; HC: 33.33 vs. 20%, *p* = 0.659; AC: 13.33 vs. 0%, *p* = 0.500; PC: 26.67 vs. 0%, *p* = 0.125, respectively).

**Figure 3:**
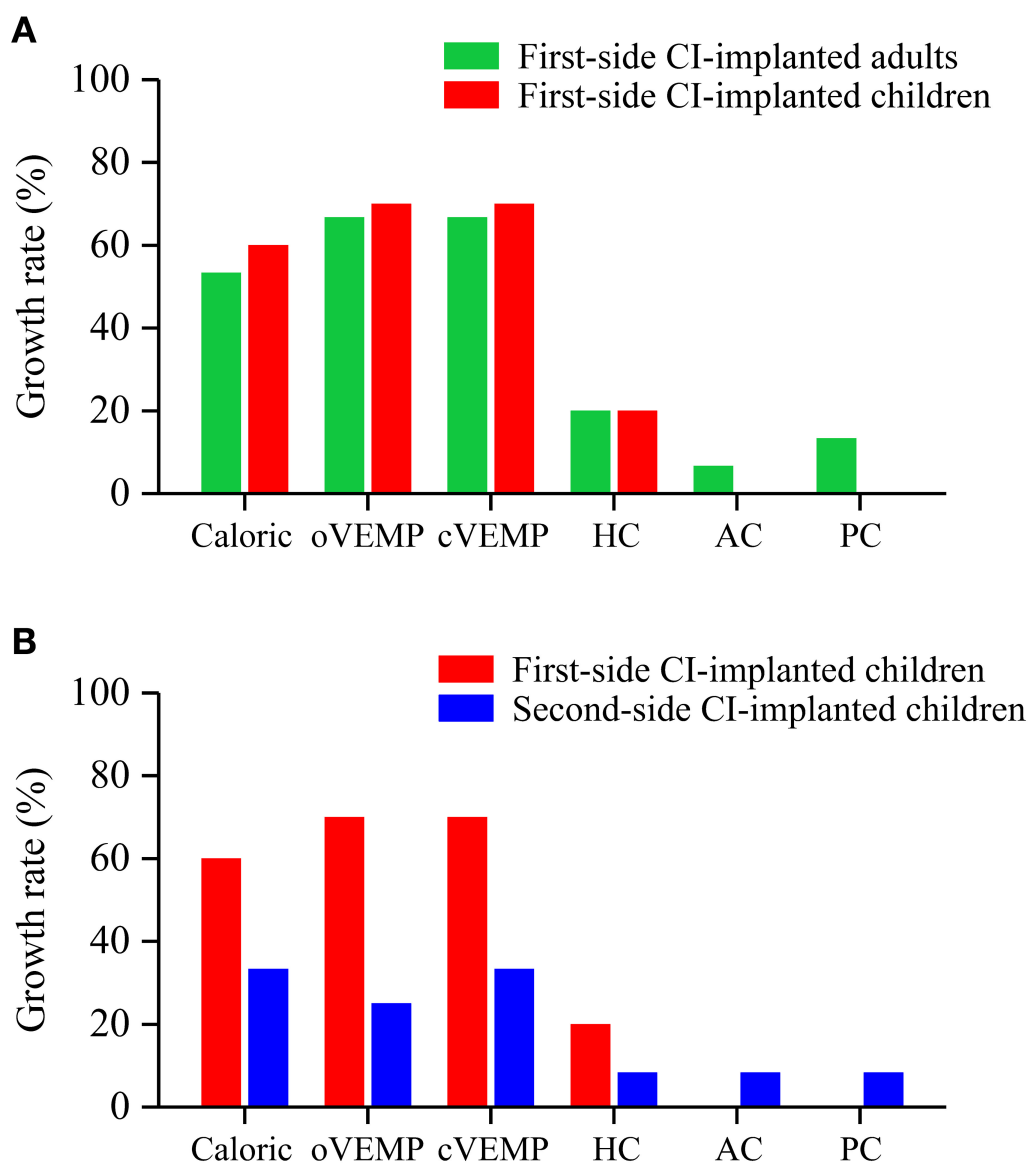
Growth rates of abnormal vestibular functions revealed by the caloric test, VEMP test and vHIT. No significant difference of growth rates was found **(A)** between first-side CI-implanted adults and children or **(B)** between first- and second-side CI-implanted children.

The growth rates in the first-side CI-implanted adults and children were 53 and 60% for caloric test, 67 and 70% for oVEMP, 67 and 70% for cVEMP, 20 and 20% for HC, 6.67 and 0% for AC, and 13.33 and 0% for PC, respectively (Figure 3A). No significant difference of the growth rates between these two groups was found (*p* > 0.05).

### Abnormal Rates of Vestibular Functions Between First- and Second-Side CI-Implanted Children

**Figure 4:**
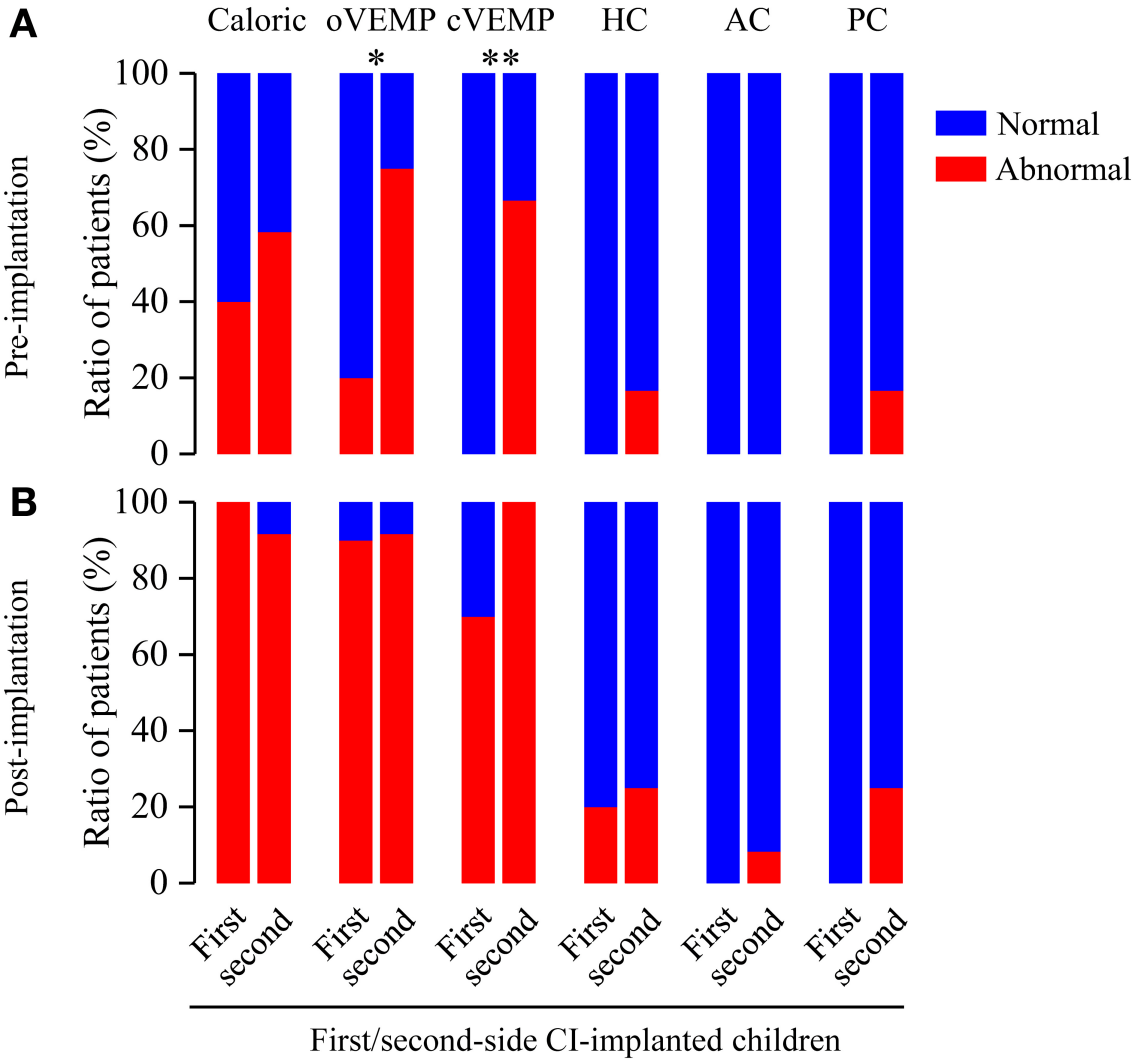
Ratio of patients with abnormal vestibular functions revealed by pre- and post-implantation caloric test, VEMP test and vHIT in first- and second-side CI-implanted children. **(A)** Higher abnormal rates for pre-implantation oVEMP and cVEMP were found in first-side CI-implanted children than in second-side CI-implanted children. **(B)** Post-implantation tests showed no significant difference of abnormal rates between these two groups. *p < 0.05, **p < 0.01.

To further assess the effect of CI approach on the vestibular functions, we analyzed the differences of abnormal rates between first- and second-side CI-implanted children. Before CI, the VEMP test showed significant differences of the abnormal rates between first- and second-side CI-implanted children (oVEMP: 20 vs. 75%, *p* = 0.030; cVEMP: 0 vs. 66.67%, *p* = 0.002, respectively) (Figure 4). No other significant difference of pre- (caloric test: 40 vs. 58.33%, *p* = 0.670; HC: 0 vs. 16.67%, *p* = 0.481; AC: 0 vs. 0%, p > 0.999; PC: 0 vs. 16.67%, *p* = 0.481) or post-implantation (caloric test: 100 vs. 91.67%, p > 0.999; oVEMP: 90 vs. 91.67%, *p* > 0.999; cVEMP: 70 vs. 100%, *p* = 0.078; HC: 20 vs. 25%, *p* > 0.999; AC: 0 vs. 8.33%, *p* > 0.999; PC: 0 vs. 25%, *p* = 0.221) abnormal rates between these two groups was found.

The growth rates in the first- and second-side CI-implanted children were 60 and 33% for caloric test, 70 and 25% for oVEMP, 70 and 33% for cVEMP, 20 and 8.33% for HC, 0 and 8.33% for AC, and 0 and 8.33% for PC, respectively (Figure 3B). No significant difference of the growth rates between these two groups was found (*p* > 0.05).

### Vertigo Questionnaires Before and After CI

The average DHI and PVSQ scores for three groups at three periods (before implantation, at day 3 after implantation and at day 30 after implantation) are shown in Table 2. The DHI scores for first-side CI-implanted adults (*p* = 0.066) and the PVSQ scores for first-side CI-implanted children (*p* = 0.206) were not significantly different among the three periods. However, we found significant differences of PVSQ scores for second-side CI-implanted children among the three periods (*p* = 0.014). The further tests showed that PVSQ score for second-side CI-implanted children at day 3 after implantation was significantly higher than that before implantation (*p* = 0.021) and that at day 30 after implantation (*p* = 0.035), respectively. There was no significant difference of the PVSQ scores between first- and second-side CI-implanted children before implantation (*p* = 0.226), at day 3 after implantation (*p* = 0.411), or at day 30 after implantation (*p* = 0.664).

**Table 2:** The pre- and post-implantation (at day 3 and at day 30) scores of Dizziness Handicap Inventory (DHI) for adults and Pediatric Vestibular Symptom Questionnaire (PVSQ) for children.

**Group**	***N***	**Scores of DHI/PVSQ**		
		**Pre-implantation**	**post-implantation (at day 3)**	**post-implantation (at day 30)**
First-side CI-implanted adults	12	18.83 ± 25.99	43.33 ± 32.84	16.00 ± 17.66
First-side CI-implanted children	8	7.00 ± 4.24	11.63 ± 4.72	8.00 ± 4.11
Second-side CI-implanted children	9	6.22 ± 9.27	14.11 ± 6.66^*^	8.22 ± 8.61^#^

*Data are expressed as mean ± standard deviation*.

* *p < 0.05 vs. pre-implantation*.

# *p < 0.05 vs. post-implantation (at day 3)*.

A Spearman's correlation test further showed a significantly positive correlation between the DHI score and the UW of the caloric test at day 30 after implantation in first- side CI-implanted adults (*r* = 0.619, *p* = 0.032).

## Discussion

CI is the preferred method of treatment for patients with severe and profound SNHL to regain hearing and speech abilities, and it has been accepted universally. However, the post-operative complications have greatly concerned clinicians and patients. Vertigo is one of the most common complications (13). The vestibule and the cochlea share a continuous membranous structure and have similar receptor cells. However, the mechanism underlying vestibular symptoms caused by CI remains unclear. A histopathological study of temporal bone specimens of CI users suggests that cochlear hydrops accompanied by saccular collapse may cause attacks of vertigo with delayed onset (14). Therefore, pre-operative and post-operative assessment of vestibular functions can be made clinically mandatory.

The abnormal rates in the caloric and VEMP tests greatly increased from pre- to post-implantation for first-side CI-implanted adults and children, indicating the negative effect of CI surgery on vestibular functions. Stultiens et al. found deteriorated vestibular functions after CI using the caloric test (15). The caloric test is based on thermal conductance which may alter after CI surgery (16) and the VEMP response may be also affected by residual blood or conductive loss in the middle ear. It should be noted that the patients had normal middle ear pressures revealed by the tympanometry before and after CI in our study, suggesting normal middle-ear conduction function. Therefore, the differences between pre- and post-operative caloric and VEMP tests may mainly reflect vestibular dysfunction resulting from CI surgery. However, we cannot completely rule out the effect of changes in thermal conductance between the external auditory canal and the labyrinth and those in the middle ear structure on the post-operative results. Moreover, there was no significant difference of the abnormal rates before and after CI in second-side CI-implanted children. This may be explained by the ceiling effect that the vestibular functions of these children already had been negatively affected by the first CI. The vHIT test showed no significant difference of the abnormal rates between pre- and post-implantation, consistent with previous findings (17). Jutila et al. reported that only 10% of patients had a reduction in VOR gain after CI (18). These findings suggest that damaged vestibular functions caused by CI may be better reflected by the caloric test compared with vHIT. Tsuji et al. analyzed the vestibular hair cells of 30 patients with Meniere's disease and found that the number of type II hair cells in patients with endolabyrinthine hydrops was significantly lower than that in the control group, whereas the numbers of type I hair cells were similar between the two groups (19). Type I hair cells (for high frequency) are located at the crest of the crista ampullaris and type II hair cells (for low frequency) are mainly located at the periphery of the crista ampullaris. Therefore, type II hair cells are closer to the perilymph space compared with type I hair cells and may be more vulnerable to endolabyrinthine hydrops. Similar to Meniere's disease, CI can also result in hydrops (14), possibly causing damage to type II hair cells. Our results are also consistent with the findings of Kuang et al. (20). Ibrahim et al. also concluded that CI surgery affects the results of caloric and VEMP tests, but not those of vHIT (17). These findings indicate that otolith and low-frequency SCC are more vulnerable to CI.

Many factors, such as age, affect the vestibular functions revealed by the VEMP test (21). With the increase of age, the hardness of the otolith structure will increase and the VEMP response rate will decrease (22). In the present study, we investigated whether the age at CI affects the vestibular functions by comparing the abnormal rates between adults and children who underwent first-side CI. The first-side CI-implanted adults and children showed similar abnormal rates and growth rates in all tests, indicating that CI damages the vestibular functions independent of age at CI. This finding may be explained by the acute injury. The abnormal rates in caloric and VEMP tests were very high within 1 month after CI (all ≥70%) and it was difficult to observe a difference between two groups. Xu et al. also reported that the response rates of oVEMP and cVEMP declined 1 month after CI (23). A follow-up study is needed to assess the long-term effects of CI on the age of implantation.

In our study, first-side CI-implanted adults and children showed abnormal vestibular functions before CI, suggesting that severe or profound hearing loss could be accompanied by vestibular dysfunction (24). Yu and Li reported that patients with sudden deafness could show vertigo (25). Meil et al. performed a series of SCC function tests before CI and found that the abnormal rate in the caloric test was 32% (26). These studies are consistent with our findings. The vestibular organs are adjacent to the cochlea anatomically, and the factors causing severe or profound hearing loss may also damage the structure and function of the vestibular systems.

With the increasing acceptance of CI, the advantages of bilateral hearing are becoming widely recognized. We found no significant difference of abnormal rates or growth rates 1 month after CI between first- and second-side CI-implanted children, indicating that unilateral and sequential bilateral CI have similar effects on vestibular functions. However, before CI, second-side CI-implanted children showed higher abnormal rates compared with first-side CI-implanted children as revealed by the VEMP test. In the present study, the second-side CI-implanted children received bilateral CI sequentially with a mean inter-implant interval of 4 years. Therefore, the vestibular dysfunction caused by unilateral (first) CI may last for a long time. The higher baseline (pre-implantation abnormal rate) in second-side CI-implanted children may possibly explain the smaller change from pre- to post-implantation (lower growth rate, not significant) compared with first-side CI-implanted children. Inconsistent with the VEMP findings, the caloric test showed no significant difference of abnormal rates not only between first- and second-side CI-implanted children before CI but also from pre- to post-implantation in the second-side CI-implanted children, suggesting that the caloric test results tend to normalize over time. Previous findings also suggest that CI may cause certain damage to the function of the saccule and utricle, revealed by cVEMP and oVEMP, respectively, and this damage can last for a long time (23). It has been further reported that the saccule, which is close to the cochlea, is the structure most vulnerable to damage from CI as most patients with CI have reduced saccular function measured by cVEMP (27, 28). The next most vulnerable structure is the utricle because of its distance from the cochlea (29). The vestibular functions are greatly damaged by both unilateral and sequential bilateral CI.

The vertigo questionnaires demonstrated no significant difference of DHI and PVSQ scores between pre- and post-implantation for unilateral CI in both adults and children. In children with bilateral CI, PVSQ scores increased significantly at day 3 post-implantation but significantly decreased at day 30, which implies that the changes may be some acute reaction to anesthesia or to middle/inner ear trauma after the surgery. These subjective performances are quite different from the objective assessment outcomes from the caloric and VEMP tests. Abouzayd et al. also showed a poor correlation between the objective vestibular outcomes and subjective symptoms (30). Katsiari et al. found that dizziness rarely persisted beyond 1 month after CI (31). Therefore, accurate diagnosis and treatment of vestibular dysfunction only by objective questionnaires is a great challenge, especially for children. A new meta-analysis showed that only 1.7% of children in contrast to 31.3% of adults had post-operative vertigo (7). The incidence of post-operative vertigo in children is far lower than that in adults, possibly related to children's poor ability to express themselves. It is difficult for children to accurately and clearly describe the symptoms of vertigo. Therefore, it is necessary to make a comprehensive and systematic assessment for vestibular functions by combining objective with subjective methods. In our study, we found a significantly positive correlation between the DHI score and the results of the caloric test at day 30 after implantation in first-side CI-implanted adults but not in children. This finding implies that, compared with children, adults with vestibular dysfunction may suffer more from postoperative vertigo. Long-term impacts on vestibular function from CI surgery, as well as the chronic electrical stimulation to the cochlea, are still to be investigated.

## Conclusions

In this study, we observed that vestibular function improved in the short term from day 3 to day 30 post-implantation. Increased abnormal rates from pre- to post-implantation in caloric and VEMP tests but not in vHIT suggest that otolith and low-frequency SCC are more vulnerable. No significant difference of abnormal rates after CI between first-side CI-implanted adults and children or between first- and second-side CI-implanted children, further indicating that alterations of vestibular function resulting from CI surgery may be independent of age at CI and CI approach (unilateral or sequential bilateral).

## Data Availability Statement

The raw data supporting the conclusions of this article will be made available by the authors, without undue reservation.

## Ethics Statement

The studies involving human participants were reviewed and approved by Anhui Provincial Hospital Ethics Committee. Written informed consent to participate in this study was provided by the participants' legal guardian/next of kin.

## Author Contributions

RG, JinS, XG, and JiaS conceived and designed the experiments. RG, YW, SW, BZ, and XG recruited participants and performed data acquisition. RG, YW, JinS, XG, and JiaS analyzed the data. All authors wrote the paper and approved the final article.

## Conflict of Interest

The authors declare that the research was conducted in the absence of any commercial or financial relationships that could be construed as a potential conflict of interest.
